# The Hospital Incident Command System: Modified Model for Hospitals in Iran

**DOI:** 10.1371/currents.dis.45d66b5258f79c1678c6728dd920451a

**Published:** 2015-03-27

**Authors:** Ahmadreza Djalali, Vahid Hosseinijenab, Mahmoudreza Peyravi, Mahmood Nekoei-Moghadam, Bashir Hosseini, Lisa Schoenthal, Kristi L. Koenig

**Affiliations:** Research Center in Emergency and Disaster Medicine and Computer Science Applied to Medical Practice (CRIMEDIM), Università del Piemonte Orientale, Novara, Italy; Department of Health, Safety and Environment, Shahid Beheshti University of Medical Sciences, Tehran, Iran; Prehospital and Disaster Medicine Centre, Sahlgrenska Academy, Gothenburg University, Gothenburg, Sweden; Department of Medical Informatic Management, Shiraz University of Medical Sciences, Shiraz, Iran; Research Center of Health Services Management and Institute for Futurology in Health, Kerman University of Medical Sciences, Kerman, Iran; Disaster Management, Natural Disaster Research Institute, Tehran, Iran; Disaster Medical Services Division, California Emergency Medical Services Authority, Rancho Cordova, California, USA; Center for Disaster Medical Sciences, University of California, Irvine, California, USA; World Association for Disaster and Emergency Medicine (WADEM)

## Abstract

Introduction: Effectiveness of hospital management of disasters requires a well-defined and rehearsed system. The Hospital Incident Command System (HICS), as a standardized method for command and control, was established in Iranian hospitals, but it has performed fairly during disaster exercises. This paper describes the process for, and modifications to HICS undertaken to optimize disaster management in hospitals in Iran. Methods: In 2013, a group of 11 subject matter experts participated in an expert consensus modified Delphi to develop modifications to the 2006 version of HICS. Results: The following changes were recommended by the expert panel and subsequently implemented: 1) A Quality Control Officer was added to the Command group; 2) Security was defined as a new section; 3) Infrastructure and Business Continuity Branches were moved from the Operations Section to the Logistics and the Administration Sections, respectively; and 4) the Planning Section was merged within the Finance/Administration Section. Conclusion: An expert consensus group developed a modified HICS that is more feasible to implement given the managerial organization of hospitals in Iran. This new model may enhance hospital performance in managing disasters. Additional studies are needed to test the feasibility and efficacy of the modified HICS in Iran, both during simulations and actual disasters. This process may be a useful model for other countries desiring to improve disaster incident management systems for their hospitals.

## Introduction

Hospitals have a key role in medical management of human impacts of mass casualty incidents and disasters. Effectiveness of hospital response to disasters requires a well established readiness before receiving a surge of casualties. Hospitals readiness comes from a set of elements, such as planning, command organization and resources.^1^

The Incident Command System was created in 1970 in the United States, by California Fire Service partners, and then was adapted by California Emergency Medical Services partners for hospital use in 1991. This adaption for hospitals was entitled the Hospital Emergency Incident Command System (HEICS) and was revised in 1992 and 1998.^2^^,^ 3 The sponsor of HEICS, the California Emergency Medical Services Authority (EMSA), dropped the “E” from HEICS with the fourth edition in 2006 because Incident Command System principles are not only applicable to emergencies- thus HEICS became HICS, the Hospital Incident Command System.^4 ^^,^HICS was updated again in 2014.^5^ Changes in the 2014 fifth edition include: greater emphasis on Incident Action Planning, updates to include addressing new threats (e.g., active shooter) and technologies (e.g., social media) and more attention to regional coordination.^5^

HICS is a standardized management system with clearly delineated and functionally based operating procedures for hospitals. It is the most commonly used model for hospital disaster management in the United States and is also used in Taiwan and Turkey.^6^^,^ 7 ^,^ 8 ^,^ 9  California EMSA is also in communication with representatives from Puerto Rico, Columbia and Japan who report their use of HICS.

In contrast to the United States where HICS originated and the human impacts of disasters is estimated as 50 people out of 1 million inhabitants in the disaster area, Iran is highly vulnerable with regard to the human impact of disasters.^10^^,^ 11 For example, in the city of Bam alone, with 100,000 inhabitants, an earthquake in 2003 left more than 30,000 people injured, and 12,000 of them were evacuated to a small number of hospitals within 1-2 days.^12^

Consistent with the national disaster management plan, and on the basis of data and experience gained from the Bam earthquake, the ministry of health in Iran considered health system preparedness to be a priority, and the HEICS/HICS was implemented nationwide.^13^^,^ 14 However, studies show fair performance of HICS in hospitals in Iran with multiple internal and external barriers to use of this system.^14^^,^ 15

Although the HICS is a flexible model and applicable in all hospitals, there are different considerations and requirements by the user hospitals, especially at the international level and in countries with diverse socio-economic backgrounds such as Iran.^15^ The lack of high performance of the HICS in Iran might be related to its incompatibility with the existing management structures in hospitals.^14^ In addition, an unpublished survey conducted by a current paper author, shows that 87% of respondents (127 managers and key personnel from 23 hospitals) believe that HICS comes from a developed country and is not fully matched to the managerial organization of hospitals in developing countries such as Iran, and doesn’t work very well during a disaster, therefore a modification of the HICS would make it more suitable and compatible.

California EMSA also supports the modification and adaptation of HICS in order to meet the organizational needs to hospitals both in the United States and internationally.

This paper describes the process and outlines the modifications to HICS that seek to optimize its applicability for managing disasters in hospitals in Iran.

## Methods

This study was conducted in 2013. HICS was modified for hospitals in Iran on the basis of the fourth edition of HICS published in 2006.^4^

A group of 11 subject matter experts participated in a modified Delphi to modify the HICS. Participants each had at least 5-years background in hospital disaster preparedness training, research and management.

The modification of HICS for Iranian hospitals was implemented on the basis of the experts’ consensus. The level of consensus was defined as 80% (9 of 11 experts).

Three rounds of modified Delphi were conducted. In the first step, the study objective, supportive documents, and first draft of a modified chart of the hospital incident command system was explained to the experts. The experts were asked if they agree with the modifications, and whether they have any comments.

While the experts’ feedback was received, the items having 85% consensus of the experts were accepted. The items having consensus level as less than 85% were modified or deleted.

Second version of the modified chart was again distributed among the experts. The experts were asked to give their opinion on new changes. The items confirmed in the first round were available to them, too. After getting the feedback of the second round, the items were accepted or deleted on the basis of consensus level as 85%, and the modified HICS-2006 for Iran was finalized and confirmed by the experts.

To comply with ethical requirements, all participants were informed they could refuse to participate or could withdraw from the study at any time. Names and personal information of the participants were kept confidential.

**Organization of the Hospital Incident Command System-2006**

HICS-2006 is composed of 69 positions, categorized in a Command group and four sections including Operations, Planning, Logistics and Finance/Administration (Figure 1).^4^

**First level organization of the Hospital Incident Command System:**
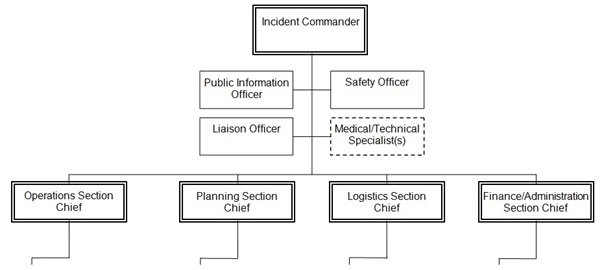

In the HICS-2006, the Command group consists of 5 positions: Incident Commander, Public Information Officer, Safety Officer, Liaison Officer, and Medical/ Technical Specialist. Depending on the type of incident, the Medical/ Technical Specialist can be an expert on chemical, radiological, legal affairs, and so forth.

The Operations Section consists of one department level management part and five branches: Staging Management, Medical Care, Infrastructure, Security, Business Continuity, and Hazardous Material.^4^

The Planning Section consists of four units: Resource, Situation, Documentation, and Demobilization Units.^4^

The Logistics Section is subdivided into two branches: Service and Support Branch.

The Finance/Administration Section consists of four units: Time, Procurement, Compensation/Claims and Cost Units.^4^

For each of the HICS positions there is a Job Action Sheet that explains the main mission and the expected tasks within immediate, intermediate and extended operational time periods; specifically, 0-2 hours, 2-12 hours, and beyond 12 hours, respectively. It also explains the demobilization/system recovery task for each position.^4^

**Managerial organization of hospitals in Iran**

In Iran, the managerial organization of hospitals varies by type of hospital. It differs among public, private, and university hospitals, also depending on the number of beds, and type and level of medical services.

Typically, a general director is at the top of the managerial organization. A chief operating manager and a chief clinical services director work with the general director.

Public information, security, and quality control officers work with the chief operating manager. In addition, all non-clinical sections such as logistics, finance, and administrative are coordinated by the chief operating manager.

All clinical departments, such as the emergency department, operating room, and internal medicine are coordinated by the chief clinical manager.

## Results

The experts who participated in this study were medical doctors (n=6), nurses (n=2) and health system specialists (n=3).

All participants were highly educated with a degree such as PhD (n=4), Doctorate of Medicine (n=4) and Master of Science (n=3).

As a modification, some of the HICS-2006 positions (69 positions) were merged; the modified HICS for Iran has 62 positions. In addition, the Command group and all 4 sections were reorganized (Figure 2).

**The Hospital Incident Command System for hospitals in Iran as modified from the HICS-2006:**
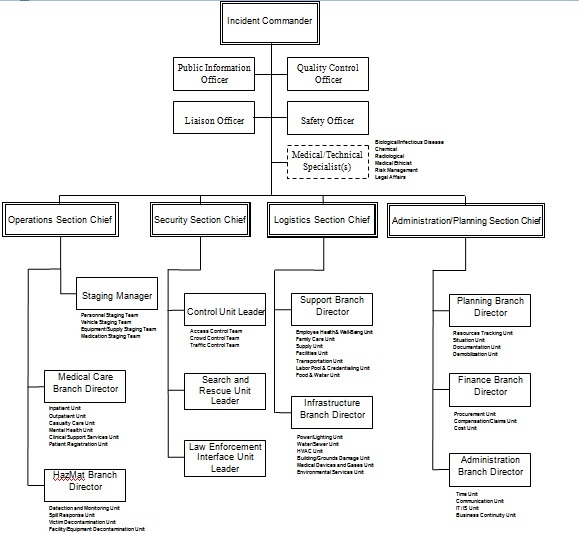

Despite these modifications, the Job Action Sheets of the HICS-2006 remained applicable for the positions of the HICS for hospitals in Iran. However, a new Job Action Sheet was defined for the “Quality Control Officer” which is the only additional position in the Iranian HICS.

**Command group**

The Command group is the core of a disaster management system. As a modification, a new position was added to the Command group of the HICS for Iran, as:

Quality Control Officer.

The main objective of this position is to assess the performance of the system, and recognize possible gaps and relevant reasons, and then report them to the Incident Commander to facilitate solving the problem. In the United States, these duties could be assigned to a Technical Specialist in Quality Improvement (Figure 1).

**Sections**

Considering the managerial structure of Iranian hospitals, the HICS sections were reorganized to: Operations, Security, Logistics, and Administration/Planning (Figure 2).

In Iranian hospitals, security is a second level position, and the security team works under the direct authority of the hospital manager/general director. As it was not practical to locate the security position in the third managerial level, the Security Branch of the Operations Section was changed to a new section: “Security.”

In most Iranian hospitals, planning activity is undertaken by the administrative section. To make the HICS suitable for Iran, the Planning Section was merged to the Administration/ Finance Section. This combined section was renamed the “Administration/ Planning Section.”

The Operations and Logistics Sections were maintained as two other sections of the HICS.

**Operations Section**

In the modified HICS for Iran, the branches “Staging Management, Medical Care, and Hazardous Material” were kept within the Operations Section; however other branches were moved as follows:

The Infrastructure Branch moved to the Logistics Section, because in Iranian hospitals these types of technical services are provided by the logistics section in daily work, therefore it is effective to keep them in the same section during disasters;

The Security Branch was moved and defined as a new section, as previously explained;

The Business Continuity Branch was moved to the Administration/Planning section. This is because in Iranian hospitals the administrative section has responsibility for planning and conducting hospital business. Therefore this function should not change in a disaster.

**Security Section**

As explained above, a new section of Security was defined in the HICS reorganization. This section consists of the:

Control Unit, including 3 teams: access control, crowd control, and traffic control.

Search and Rescue Unit

Law Enforcement Interface Unit

Despite this reorganization, there is no change in the actions of the Security Section, considering the Job Action Sheets of the original HICS-2006.

**Logistics Section**

The following changes were considered for the modification of the Logistics Section:

The Service Branch, except the Staff Food and Water Unit, moved to the Administration/ Planning Section, to be placed in the Administration Branch. The reason for this movement is that communication, information technology (IT) and information services (IS) are usually under the administration department, in Iranian hospitals. It is more reasonable to refer these activities, during disasters, to a group who performs these functions in their daily jobs.

The “Staff Food and Water” Unit remained in the Logistics Section, but was placed as a unit of the Support Branch.

The Infrastructure Branch was moved from the Operations Section to the Logistics Section. The reason of this movement was previously explained in the Operations Section.

Furthermore, the “Food Services” Unit was moved to the Support Branch, because it is more suitable to the Support Branch, considering the organization of Iranian hospitals.

In Iran, a single hospital unit usually manages both medical devices and medical gases; therefore these units were merged as the “Medical Devices and Gases Unit”.

The Support Branch was kept in the Logistics Section, with a new unit as “Food and Water.” This unit is a combination of the “Staff Food and Water Unit” from the Service Branch, and the “Food Service Unit” from the Infrastructure Branch. This new unit is responsible for provision of food and drinking water to staff, patients, families and visitors.

**Administration/Planning Section**

As previously explained, this section was established through merging the Planning Section with the Finance/Administration Section. The section was organized into 3 branches:

Planning Branch, consisting of 4 units:

Resources Tracking Unit, which is responsible to track both personnel and materials;

Situation Unit, which is responsible to track both patients and beds;

Documentation Unit;

Demobilization Unit.

The roles and responsibilities of the Planning Branch and relevant units are same as delineated in the Job Action Sheets of the whole Planning Section of the HICS-2006.

Finance Branch

Considering the HICS-2006 Job Action Sheets, the following units were placed under the Finance Branch:

Procurement Unit

Cost Unit

Compensation/Claims Unit

Administration Branch consists of four units. The following units were placed under the “Administration Branch,” because these units manage the administrative office of a hospital during daily work, therefore it is better to keep them in the same function during disasters.

Time Unit

Communications Unit (moved from the Logistics Section, Service Branch)

IT/IS Unit (moved from the Logistics Section, Service Branch)

Business Continuity Unit

The “Business Continuity Unit” was condensed into a smaller unit in this phase to make the hospitals familiar with its function during disasters. It may be reorganized to a complete unit, as part of HICS for Iran, in the future.

## Discussion

This study was undertaken to modify the HICS-2006 for Iran hospitals. The experts recommended multiple changes to HICS for hospitals in Iran. The sections, branches and positions were redesigned, merged, and moved to make the HICS more suitable for Iranian hospitals. This type of modification may lead to improved disaster management. Expert opinion and experience and knowledge gained from major events and disasters, such as terrorist attacks in the USA and Japan, and biological outbreaks led the researchers to seek improvements in disaster management. The sponsoring organization of HICS regularly updates and modifies this system to remove possible gaps and deficiencies in its organization and activities and to enhance its performance.^3^^,^ 4 ^,^ 5 ^,^ 6 However, HICS may need modification in some countries depending on existing infrastructures. Further studies should evaluate the efficacy of this modified HICS during exercises and actual disasters in Iran.

A quality control position was added to the HICS modified for Iranian hospitals. The control of hospital performance and quality in responding to disasters is essential to detect and eliminate gaps and weaknesses in hospital disaster management functions. These actions may improve the clinical outcomes of the casualties.^16^^,^ 17 The Quality Control Officer provides real-time analysis of HICS performance for the Incident Commander, while the hospital focuses on management of disaster victims. It is expected that this new position will be effective in enhancing HICS performance in Iranian hospitals, however this theory must be tested in future studies.

In this study, three non-medical branches of the Operations Section were moved to other sections to make the Operations Section function lighter, quicker and more focused on the medical mission. The human impact of disasters in Iran is high,^11^^,^ 18 and the workload rapidly escalates in situations where hospitals receive hundreds of casualties.^12^ Despite the threats, prior studies show low levels of preparedness and response performance in Iranian hospitals, in terms of medical operations and functions.^14^^,^ 19 ^,^ 20 These changes in the Operations Section will enable enhanced use of all capacities to provide medical services to the casualties, which may decrease mortality and morbidity. The impact of this modification also needs to be evaluated by future studies.

Another major modification of HICS for Iran is the addition of Security as a new section. As in many countries, emergency department crowding is a major issue in Iran,^21^ especially during disasters. Also, lack of hospital access due to a crowded transportation infrastructure is a major challenge for victims’ access to hospitals during disasters.^12^ Despite this known factor, hospital preparedness with regards to security including control of access and control of traffic is limited.^22^^,^ 23

Furthermore, Iranian hospital buildings are vulnerable to disasters^19^^,^ 24 and may suffer both structural and non-structural damage, for example, during an earthquake. This hazard dictates that well prepared search and rescue teams are needed for Iranian hospitals.

Adding Security as a section in the HICS will likely result in better performance of the relevant operations, such as control of access, regulating traffic, and search and rescue operations during disasters. As with other modifications, this change should be evaluated by other studies.

In this study, the Infrastructure Branch was moved to the Logistic Section. The subjects of the Infrastructure Branch Units could be classified as structural and non-structural elements of hospitals,^25^ which are non-medical technical services. These elements are defined as technical/logistics services at Iranian hospitals. To maintain these services in the Logistics Section may facilitate a safe hospital strategy as well as making the Operations Section free of non-medical services while the hospital manages a disaster.

Planning is a core element of disaster management, and it may be considered as a part of preparedness.^1^ In the preparedness phase, the hospital manager coordinates planning activities as a part the administrative section. The Administrative/Planning Section provides a cost-benefit for Iranian hospitals through preventing duplication of work. It also enhances the efficiency of planning function, before and during disasters.

## Conclusion

This modified HICS is more suitable to the managerial organization of hospitals in Iran, therefore it may enhance performance of hospitals during disasters and reduce mortality and morbidity. Additional studies are needed to test the feasibility and outcomes associated with the modified HICS in Iran, both during simulations and real disasters.

The approach described in this paper may be used by other countries with high human impacts of disasters that are looking for a model for an incident management system to implement within their hospital systems.

## Competing Interests

The authors have declared that no competing interests exist.
